# Experimental and theoretical interpretation of the magnetic behavior of two Dy(iii) single-ion magnets constructed through β-diketonate ligands with different substituent groups (–Cl/–OCH_3_)†

**DOI:** 10.1039/c8ra06240d

**Published:** 2018-08-20

**Authors:** Sheng Zhang, Wenjiao Mo, Jiangwei Zhang, Haipeng Wu, Min Li, Xingqiang Lü, Bing Yin, Desuo Yang

**Affiliations:** College of Chemistry and Chemical Engineering, Baoji University of Arts and Sciences Baoji 721013 China zhangsheng19890501@163.com yangdesuo@163.com; Key Laboratory of Synthetic and Natural Functional Molecule Chemistry of Ministry of Education, College of Chemistry and Materials Science, Northwest University Xi'an Shaanxi 710069 China rayinyin@nwu.edu.cn; School of Chemical Engineering, Shaanxi Key Laboratory of Degradable Medical Material, Northwest University Xi'an 710069 Shaanxi China; State Key Laboratory of Catalysis & Gold Catalysis Research Center, Dalian Institute of Chemical Physics, Chinese Academy of Sciences (CAS) China

## Abstract

Two Dy(iii) single-ion magnets, formulated as [Dy(Phen)(Cl-tcpb)_3_] (Cl-1) and [Dy(Phen)(CH_3_O-tmpd)_3_] (CH_3_O-2) were obtained through β-diketonate ligands (Cl-tcpb = 1-(4-chlorophenyl)-4,4,4-trifluoro-1,3-butanedione and CH_3_O-tmpd = 4,4,4-trifluoro-1-(4-methoxyphenyl)-1,3-butanedione) with different substituent groups (–Cl/–OCH_3_) and auxiliary ligand, 1,10-phenanthroline (Phen). The Dy(iii) ions in Cl-1 and CH_3_O-2 are eight-coordinate, with an approximately square antiprismatic (SAP, *D*_4d_) and trigonal dodecahedron (*D*_2d_) N_2_O_6_ coordination environment, respectively, in the first coordination sphere. Under zero direct-current (dc) field, magnetic investigations demonstrate that both Cl-1 and CH_3_O-2 display dynamic magnetic relaxation of single-molecule magnet (SMM) behavior with different effective barriers (*U*_eff_) of 105.4 cm^−1^ (151.1 K) for Cl-1 and 132.5 cm^−1^ (190.7 K) for CH_3_O-2, respectively. As noted, compound CH_3_O-2 possesses a higher effective barrier than Cl-1. From *ab initio* calculations, the energies of the first excited state (KD_1_) are indeed close to the experimental *U*_eff_ as 126.7 cm^−1^*vs.* 105.4 cm^−1^ for Cl-1 and 152.8 cm^−1^*vs.* 132.5 cm^−1^ for CH_3_O-2. The order of the calculated energies of KD_1_ is same as that of the experimental *U*_eff_. The superior SIM properties of CH_3_O-2 could have originated from the larger axial electrostatic potential (ESP_(ax)_) felt by the central Dy(iii) ion when compared with Cl-1. The larger ESP_(ax)_ of CH_3_O-2 arises from synergic effects of the more negative charge and shorter Dy–O distances of the axial O atoms of the first sphere. These charges and distances could be influenced by functional groups outside the first sphere, *e.g.*, –Cl and –OCH_3_.

## Abstract 2

Further studies from the viewpoint of electrostatic potential demonstrate that the larger axial electrostatic potential (ESP) felt by the central Dy (iii) ion of CH_3_O-2 is responsible for its better SIM property when compared with Cl-1.

## Graphical abstract

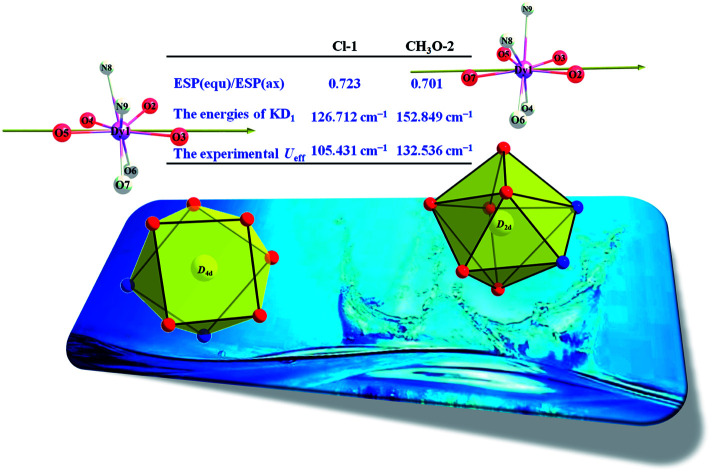

## 1. Introduction

Since the first single-molecule magnet (SMM), Mn_12_Ac, was discovered in the 1990s,^1^ the search for improved properties, in particular the enhanced relaxation barrier (*U*_eff_) and blocking temperature (*T*_B_), has advanced the development of new compounds with magnetic properties customized by the coordination environment.^2^ The relevant explorations focus on mononuclear lanthanide compounds, especially single Dy-center systems, owing to the intrinsic strong spin–orbit coupling and large magnetic anisotropy of lanthanide ions.^3^ Furthermore, the relatively simple structure is convenient for chemists to improve the understanding of the magneto-structural correlation by combining with *ab initio* calculations.^4^ The number of mononuclear lanthanide SMMs or single-ion magnets (SIMs) derived from the above approach, following significantly slow relaxations of the magnetization, has grown significantly. An equatorially coordinated triangular geometry for *C*_3_,^5^ square antiprismatic systems for *D*_4d_,^6^ sandwich-type systems for *D*_8d_ or *D*_8h_,^7^ the pentagonal bipyramidal local geometry for *D*_5h_,^4*a*,4*b*,8^ linear 2-coordinated systems for *D*_∞h_^9^ and some systems possessing high charge distribution symmetry,^10^ are given different priorities in lanthanide SIM construction. The research reveals that the single-ion magnetic anisotropy of lanthanide ions is extremely sensitive to the subtle changes in the ligand and the local geometrical symmetry. The electrostatic potential distribution around the magnetic center can be regulated by the amount of solvent,^11^ the anion ligands,^12^ the lattice/coordinated solvents,^13^ the pH values of the solution systems,^14^ or the counter ions,^15^ on the basis of weakening or strengthening the electron density.^16^ Interestingly, other functional groups from ligands outside the first sphere could also effectively influence the electron density of the first-sphere atoms and thus affect the SIM properties, which should also be considered in the rational design of promising molecular magnets.^4*d*^

Finding a feasible strategy to regulate and control the electrostatic environment around the metal centers and the single-ion magnetic anisotropy in SIMs would provide an active direction for understanding the magneto-structural correlation in depth and developing magnetic materials with high *U*_eff_ and blocking temperature (*T*_B_). Herein, two β-diketonate ligands with different functional groups were chosen for the following reasons: the classical β-diketonate ligands with different functional groups usually coordinate with metal ions in bidentate chelating modes in SIMs, which is beneficial for discussing the influence of the electron density.^8^ Fortunately, with the introduction of a capping ligand, two mononuclear compounds, [Dy(Phen)(Cl-tcpb)_3_] (Phen = 1,10-phenanthroline, Cl-tcpb = 1-(4-chlorophenyl)-4,4,4-trifluoro-1,3-butanedione, Cl-1) and [Dy(Phen)(CH_3_O-tmpd)_3_] (CH_3_O-tmpd = 4,4,4-trifluoro-1-(4-methoxyphenyl)-1,3-butanedione, CH_3_O-2), were obtained through solution reaction. The Dy(iii) ion in compound Cl-1 has an approximately square antiprismatic (SAP) N_2_O_6_ coordination environment, while CH_3_O-2 has a trigonal dodecahedron (*D*_2d_) configuration. Magnetic characterization indicates that the subtle changes in the different substituent groups from the β-diketonate ligand results in great differences in the coordination environment and dramatically alters the relaxation behaviors of Cl-1 and CH_3_O-2. To further understand the different magnetic behaviors of Cl-1 and CH_3_O-2, *ab initio* calculations were also performed to explore the magnetic anisotropies of the central Dy(iii) ions. A preliminary analysis on the electrostatic potential felt by the Dy(iii) ion is utilized to identify the effect of ligands on its magnetic anisotropy. Expectedly, other functional groups outside the first sphere could also effectively influence the electron density of the first sphere atoms and thus affect the SIM properties.

## 2. Experimental

### 2.1 Materials and instruction

All the materials and reagents were obtained commercially without further purification. The FT-IR spectra were recorded in the range of 400–4000 cm^−1^ using KBr pellets on an EQUINOX55 FT/IR spectrophotometer. Elemental analysis (C, H, N) was implemented on a Perkin-Elmer 2400 CHN elemental analyzer. The phase purity of the bulk or polycrystalline samples were confirmed by powder X-ray diffraction (PXRD) measurements executed on a Rigaku RU200 diffractometer at 60 kV, 300 mA, and Cu K*α* radiation (*λ* = 1.5406 Å), with a scan speed of 5° min^−1^ and a step size of 0.02° in 2*θ*. Diffuse reflectance spectra were obtained by a U-41000 spectrophotometer applying BaSO_4_ powder as a 100% reflectance reference. Magnetic measurements were performed in the temperature range of 1.8–300 K with an applied field of 1000 Oe, using a Quantum Design MPMS-XL-7 SQUID magnetometer on polycrystalline samples. The diamagnetic corrections for the compounds were estimated using Pascal's constants. Alternating current (ac) susceptibility experiments were performed using an oscillating ac field of 2.0 Oe at ac frequencies ranging from 1 to 1000 Hz. The magnetization was measured in the field range 0–7 T.

### 2.2 Synthesis and characterization of the lanthanide compounds

All chemicals were obtained from commercial sources and were used as received without further purification.

#### Synthesis of [Dy(Phen)(Cl-tcpb)3] (Cl-1)

A methanol solution (10 mL) of Dy(NO_3_)_3_·6H_2_O (0.1 mmol, 0.0456 g) and Phen (0.1 mmol, 0.0200 g) was added to a solution of Cl-tcpb (0.3 mmol, 0.0753 g) and KOH (0.3 mmol, 0.0168 g) in 10 mL of dichloromethane under stirring. The resultant solution was filtered and allowed to stand undisturbed at room temperature for three weeks. Block crystals of Cl-1 were obtained in 37% yield (based on the Dy(iii) salt). Anal. Calcd for C_45_H_32_DyF_9_N_2_O_6_: C, 52.42; H, 3.11; N, 2.72. Found: C, 52.77; H, 3.32; N, 2.53. IR (KBr): 3077 (w), 1645 (s), 1623 (m), 1553 (s), 1564 (m), 1453 (w), 1401 (m), 1334 (w), 1299 (s), 1168 (m), 1111 (s), 1043 (w), 1009 (m), 923 (m), 833 (m), 801 (w), 775 (w), 722 (w), 677 (w), 611 (m), 575 (w), 487 (m), 439 cm^−1^ (w).

#### Synthesis of [Dy(Phen)(CH3O-tmpd)3] (CH_3_O-2)

A similar synthetic procedure to that for Cl-1 was used to synthesize CH_3_O-2, except that Cl-tcpb (0.3 mmol, 0.0753 g) was replaced by CH_3_O-tmpd (0.3 mmol, 0.0738 g). Finally, pink block crystals were obtained after three weeks in 51% yield (based on the Dy(iii) salt). Anal. Calcd for C_47_H_36_DyF_9_N_2_O_7_: C, 52.50; H, 3.35; N, 2.61. Found: C, 52.38; H, 3.52; N, 2.82. IR (KBr): 3092 (w), 1621 (s), 1589 (s), 1567 (s), 1533 (s), 1462 (m), 1401 (m), 1343 (w), 1302 (m), 1166 (m), 1145 (m), 1054 (w), 1022 (m), 921 (w), 846 (m), 766 (m), 755 (w), 732 (w), 681 (m), 587 (m), 572 (m), 481 (w), 443 (w), 409 (m) cm^−1^.

### 2.3 X-ray single-crystal diffraction analysis

The single crystal X-ray experiment was performed on an Agilent Xcalibur Eos Gemini diffractometer using graphite-monochromatized Cu K*α* radiation (*λ* = 1.5418 Å). The data integration and reduction were processed with the CrysAlisPro software. Absorption correction based on multi-scans was performed using the SADABS program.^17^ The structures were solved by the direct method and refined by means of full-matrix least-squares procedures on *F*^2^ with the SHELXL program.^18^ All non-hydrogen atoms were refined anisotropically. Other details of crystal data, data collection parameters, and refinement statistics are given in Table S1.† The selected bond lengths and angles are listed in Table S2.†

### 2.4 Theoretical methods and computational details

Multiconfigurational *ab initio* calculations, including spin–orbit coupling (SOC), were performed on the experimental structures of 1 and 2 to explore their magnetic anisotropy. This type of calculation includes two steps:^19^ (1) a set of spin eigenstates, were obtained by the state-averaged (SA) CASSCF method;^20^ (2) the low-lying SOC states, *i.e.*, Kramers doublets (KD) herein, were obtained by state interaction, which is the diagonalization of the SOC matrix in the space spanned by the spin eigenstates from the first step. In the CASSCF step, the active space consisted of 9 electrons in 7 orbitals and all the spin eigenstates of 21 sextets were included. Due to the hardware limitations, other highly excited quartets and doublets were not considered. The state interaction step was performed by the RASSI-SO module^21^ with the SOC integrals from the AMFI method.^22^ The ANO-RCC basis sets,^23–25^ including VTZP for Dy, VDZ for C and H as well as VDZP for other atoms, were used. All the calculations were carried out with the MOLCAS@UU, a version of MOLCAS 8.0 (ref. 26 and 27) which is freely distributed for academic users. The SINGLE_ANISO module,^28,29^ developed by Chibotaru *et al.*, was used to obtain the g-tensors, transition magnetic moments and other parameters characterizing the magnetic anisotropy.

## 3. Results and discussion

### 3.1 Crystal structures

**Fig. 1:**
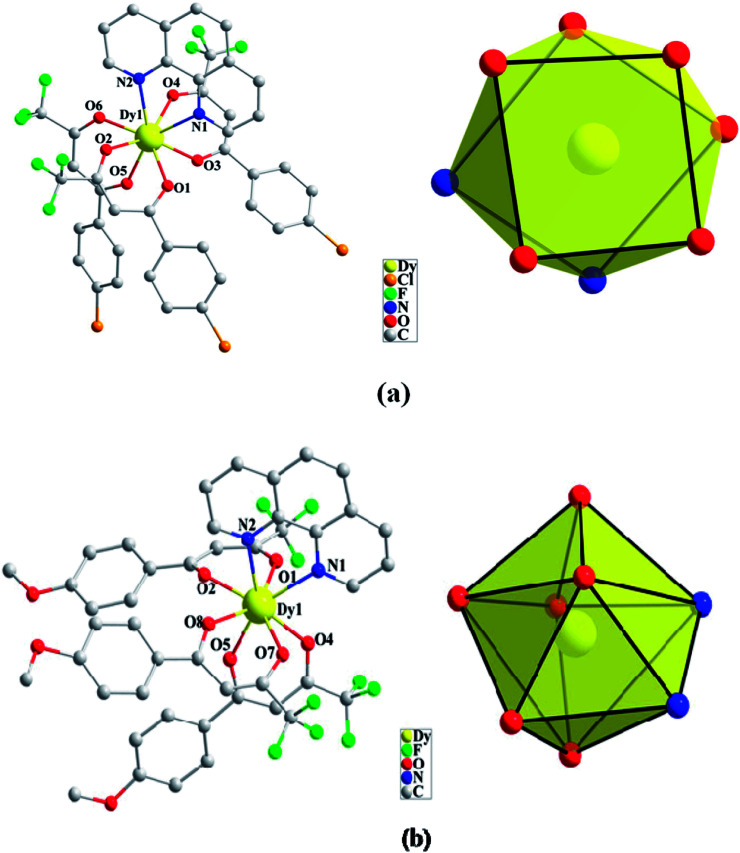
Coordination environments of Cl-1 (a) and CH_3_O-2 (b) and the local coordination geometries of the Dy(iii) ions of Cl-1 (a) and CH_3_O-2 (b); hydrogen atoms were omitted for clarity.

Compounds Cl-1 and CH_3_O-2 are crystallized in the triclinic space group *P*-1. Cl-1 and CH_3_O-2 have N_2_O_6_ coordination environments. Each Dy(iii) ion is surrounded by three negative-ion ligands and a neutral capping ligand (Phen), as shown in Fig. 1. The Dy–O distances are from 2.319 (7) to 2.369 (6) Å in Cl-1 and from 2.305 (7) to 2.350 (5) Å in CH_3_O-2. Additionally, the Dy–N distances are 2.544 (8) Å and 2.586 (8) Å in Cl-1 as well as 2.554 (6) Å and 2.571 (6) Å in CH_3_O-2. By using the SHAPE 2.1 software, the configurations of Dy(iii) ions in Cl-1 and CH_3_O-2 were calculated (Table S2†), indicating that the compounds Cl-1 and CH_3_O-2 belong to an approximately square-antiprismatic (SAP, *D*_4d_) and trigonal dodecahedron (*D*_2d_) configuration, respectively.^30^ The shortest interdinuclear Dy(iii)⋯Dy(iii) distances are 7.911 (6) Å in Cl-1 and 10.742 (18) Å in CH_3_O-2, respectively.

**Fig. 2:**
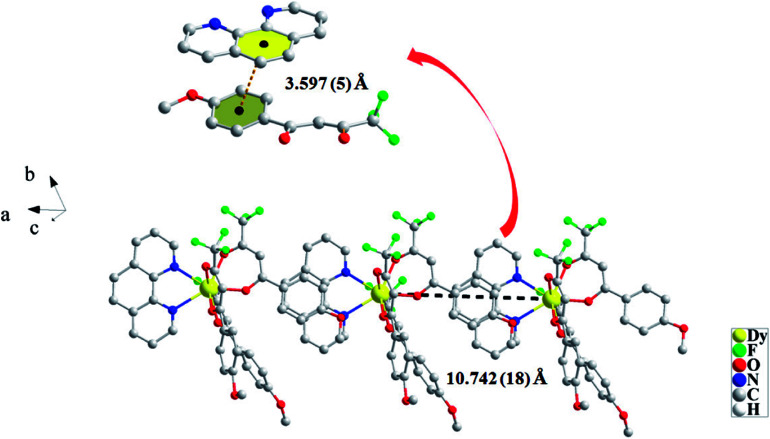
Packing arrangement between two neutral molecules to form a 1D supramolecular chain in CH_3_O-2.

For CH_3_O-2, the neutral molecules are assisted by weak ð–ð stacking between the parallel interlayer (Fig. 2) to generate a 1D supramolecular chain, and the centroid distance is 3.597 (5) Å, from a slipped stacking and leading to the Dy(iii)⋯Dy(iii) distance of 10.742 (18) Å. However, there is no obvious ð–ð stacking or hydrogen bonding interaction in compound Cl-1.

**Scheme 1:**
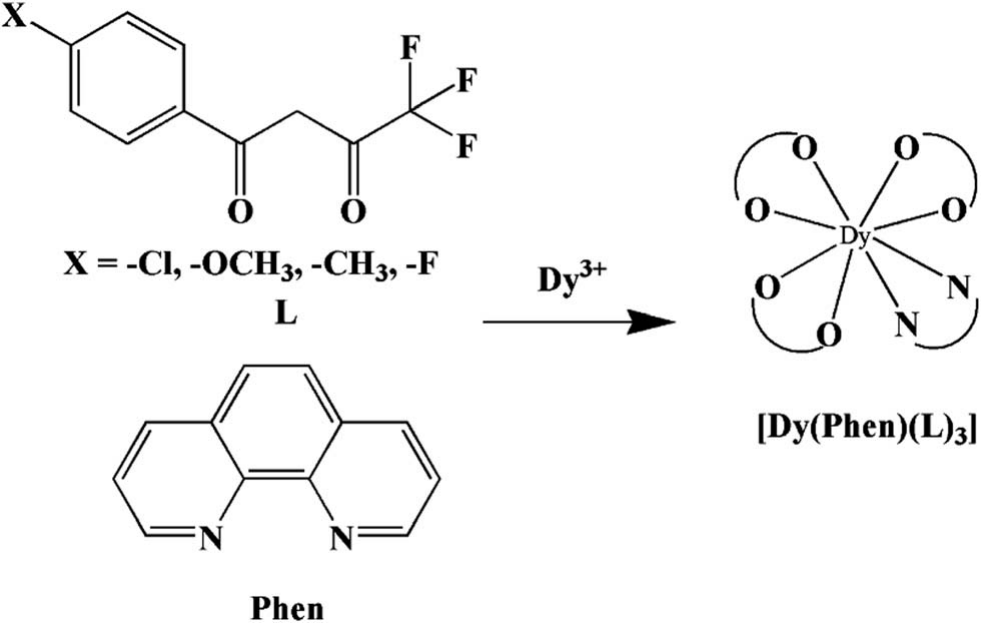
The synthetic process for compounds with molecular formula Dy(Phen)(L)_3_.

In our previous works, the β-diketonate ligands with different substituent groups (–F/–CH_3_) were employed to obtain a series of mononuclear Dy(iii) compounds (Scheme 1). For CH_3_-4 (solvent) and CH_3_-3, the latter has a trigonal dodecahedron (*D*_2d_) configuration of Dy(iii) ions, while CH_3_-4 (solvent) shows an approximately square antiprismatic (SAP, *D*_4d_) N_2_O_6_ coordination environment of Dy(iii) ions. The uncoordinated 1,4-dioxane molecules exist in CH_3_-4 (solvent). Interestingly, the compounds above have weak interactions between the neutral molecules. In CH_3_-3, the neutral molecules are assisted by weak π⋯π stacking between the parallel interlayer, and the centroid distance is 3.767 (6) Å, belonging to a slipped stacking and leading to the Dy(iii)⋯Dy(iii) distance of 9.193 (5) Å. In CH_3_-4 (solvent), the neutral molecules are connected by weak C(171)–H(117)⋯O(7) interactions, leading to the Dy(iii)⋯Dy(iii) distance of 18.853 (2) Å. F-5 belongs to an approximately SAP configuration. For Cl-1, CH_3_-4 (solvent) and F-5, the CH_3_-4 (solvent) is more inclined toward the SAP configuration, calculated by utilizing the SHAPE 2.1 software. Cl-1 has the closest distance between the neutral molecules. In CH_3_O-2 and CH_3_-3, the former has the smaller deviation relative to a trigonal dodecahedron (*D*_2d_) configuration. The longer distance between the neutral molecules can be observed in CH_3_O-2. It is a remarkable fact that the maximum average Dy–N bond length is 2.563 Å (2) in CH_3_O-2. F-5 and CH_3_O-2 show similar average Dy–O bond lengths, which are smaller than Cl-1, CH_3_-3 and CH_3_-4 (solvent). The different types of weak interactions between the neutral molecules, configurations and bond lengths would result in different magnetic behaviors.

### 3.2 Magnetic properties

**Fig. 3:**
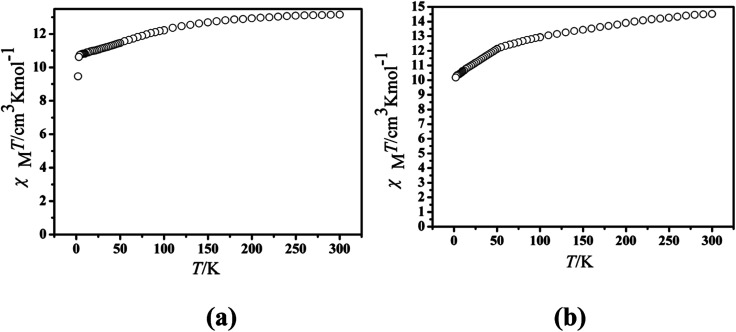
Temperature dependence of the *χ*_M_*T* product at 1000 Oe for compounds Cl-1 (a) and CH_3_O-2 (b).

The magnetic experiments of Cl-1 and CH_3_O-2 were performed on polycrystalline samples. PXRD results of Cl-1 and CH_3_O-2 support the pure state of the bulk materials (Fig. S1†). The values of *χ*_M_*T* of Cl-1 and CH_3_O-2 are 13.16 cm^3^ mol^−1^ K and 14.51 cm^3^ mol^−1^ K at room temperature, respectively, which are close to the free-ion value of 14.17 cm^3^ mol^−1^ K for a single Dy(iii) ion (^6^*H*_15/2_, *S* = 5/2, *L* = 5, *J* = 15/2, *g* = 4/3) (Fig. 3).^13^ When cooled, the *χ*_M_*T* curves for compound Cl-1 decreased slowly in the range from 300 to 100 K. Subsequently, the *χ*_M_*T* products decreased sharply below 100 K to the minimum of 9.46 cm^3^ mol^−1^ K for Cl-1 and 11.75 cm^3^ mol^−1^ K for CH_3_O-2 at 1.8 K. In CH_3_O-2, on lowering the temperature, the *χ*_M_*T* product decreased gradually and more rapidly below 50 K. These behaviors could be ascribed to crystal field splitting, particularly the progressive quenching of excited Dy(iii) Stark sublevels and/or weak intermolecular dipole–dipole effects.^32^

**Fig. 4:**
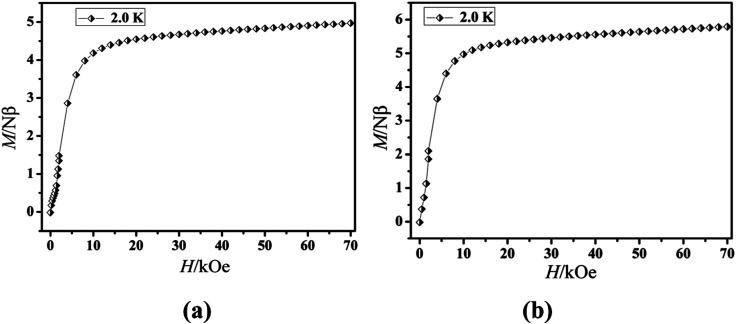
*M vs. H* plots at 2.0 K for compounds Cl-1 (a) and CH_3_O-2 (b).

**Fig. 5:**
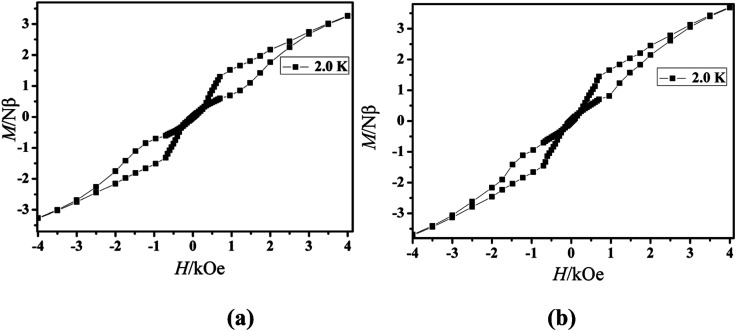
*M versus H* data of Cl-1 (a) and CH_3_O-2 (b) at 2.0 K.

The magnetization of the two compounds from zero dc field to 70 kOe at different temperatures is shown in Fig. 4. The magnetization of Cl-1 and CH_3_O-2 at 2 K increased upon application of an external field to a maximum of 4.97 Nβ and 5.79 Nβ. The maximum values in Cl-1 and CH_3_O-2 at 7 T largely deviate from the expected saturation point of 10 Nβ, consisting of the magnetic anisotropy and crystal field effects at the dysprosium center, which dispel the 16-fold degeneration of the ^6^*H*_15/2_ ground state.^33^ The *M versus H* data exhibit obvious butterfly-shaped hysteresis loops at 2 K for Cl-1 and CH_3_O-2 (Fig. 5), indicating the fast zero-field relaxation between the two ground states.

**Fig. 6:**
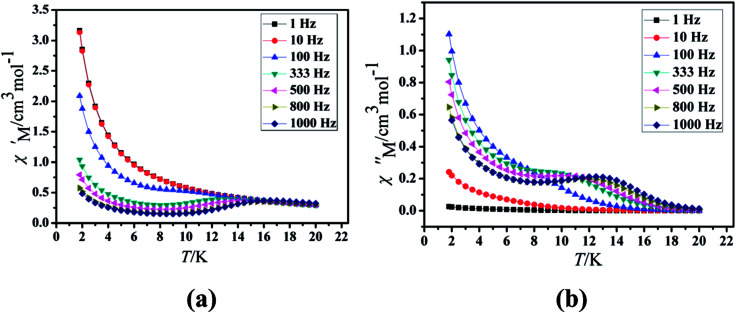
Temperature dependence of the in-phase (*χ*′, a) and out-of-phase (*χ*′′, b), respectively, of the ac susceptibility for Cl-1 under the zero-dc field.

**Fig. 7:**
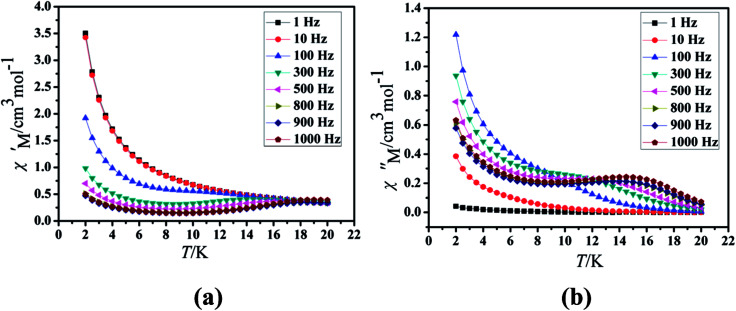
Temperature dependence of the in-phase (*χ*′, a) and out-of-phase (*χ*′′, b), respectively, of the ac susceptibility for CH_3_O-2 under the zero-dc field.

Under the oscillating field of 3.5 Oe, the zero-field AC susceptibility experiments were determined in the range of 1.8–20 K and at frequencies of 1, 10, 100, 333, 500, 800 and 1000 Hz in Cl-1. However, for CH_3_O-2, zero-field AC susceptibilities were measured in the range of 2–18 K and at frequencies of 1, 10, 100, 300, 500, 800, 900 and 1000 Hz. Both in-phase (*χ*′) and out-of-phase (*χ*′′) susceptibilities in compound Cl-1 and CH_3_O-2 showed significant temperature dependence peaks at a relatively high-temperature range (Fig. 6 and 7), which clearly indicates the slow relaxation of magnetization. When cooled, *χ*′ and *χ*′′ increased again at lower temperatures; such a situation could be due to the emergence of quantum tunneling of magnetization (QTM) without an extra dc field, which often occurs in Ln(III)-based SMMs or SIMs (Table 1).^34^

**Table 1:** The notable examples of DyN_2_O_6_ mononuclear dysprosium compounds, namely [Dy(Phen)(L)_3_], based on the *β*-diketonate ligands with different substituent groups (–CI, –CH_3_,–CH_3_O,–F) at the 4-position

L	*d*_Dy–O_	*d*_Dy–N_	*d*_Dy⋯Dy_	Symmetry	*U*_eff_ (K)	Ref.	Name
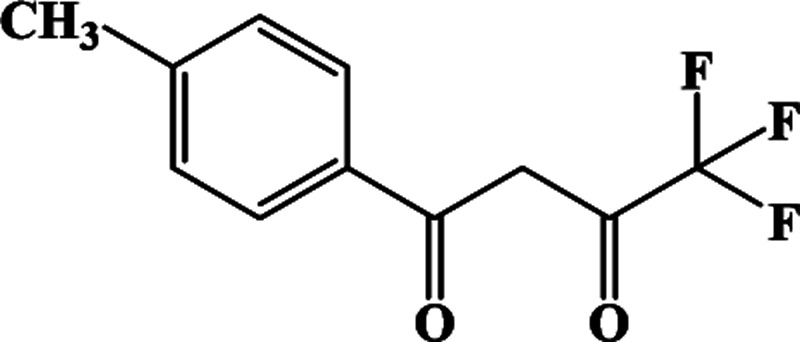	2.337 (2)	2.538 (3)	9.355 (11)	*D*_2d_	63.56 K/67.05 K	13*b*	CH_3_-3
					(0 Oe)		
					118.50 K (1200 Oe)		
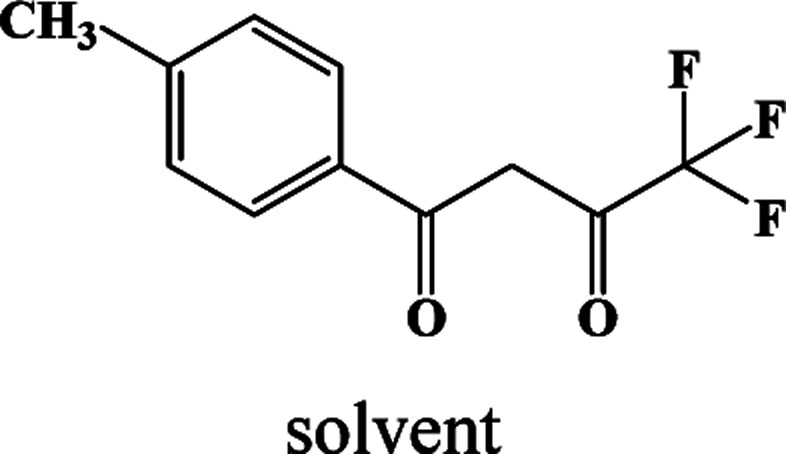	2.329 (4)	2.553 (5)	9.193 (10)	*D*_4d_	102.82 K/95.88 K	13*b*	CH_3_-4 (solvent)
					(0 Oe)		
					164.55 (1200 Oe)		
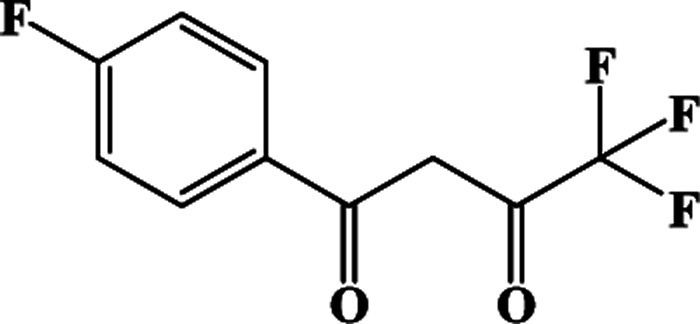	2.311 (3)	2.579 (3)	9.679 (8)	*D*_4d_	91.70 K (1200 Oe)	31	F-5
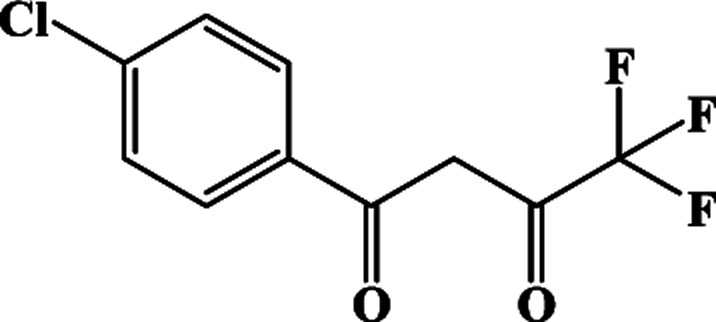	2.339 (6)	2.562 (8)	7.911 (6)	*D*_4d_	151 (0 Oe)	Here	Cl-1
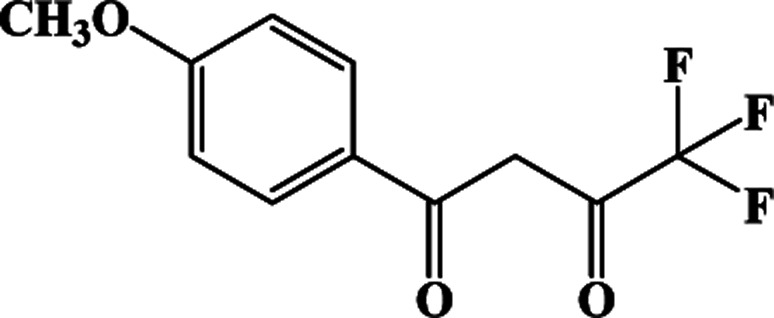	2.322 (5)	2.563 (6)	10.742 (13)	*D*_2d_	190.7 (0 Oe)	Here	CH_3_O-2

**Fig. 8:**
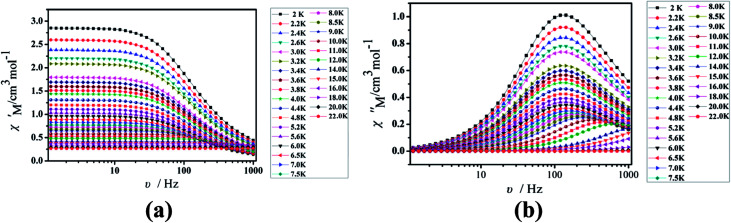
Frequency dependence of the in-phase (*χ*′, a) and out-of-phase (*χ*′′, b) of the ac susceptibility for Cl-1 under the zero-dc field.

**Fig. 9:**
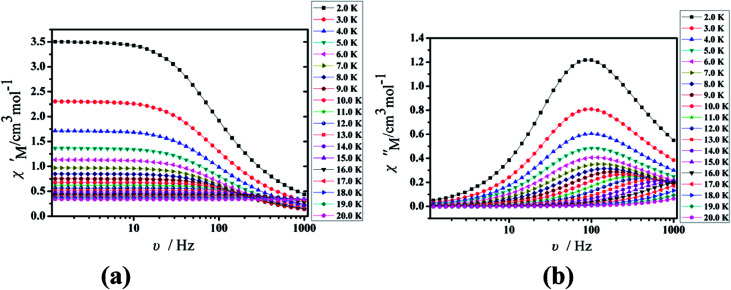
Frequency dependence of the in-phase (*χ*′, a) and out-of-phase (*χ*′′, b) of the ac susceptibility for CH_3_O-2 under the zero-dc field.

**Fig. 10:**
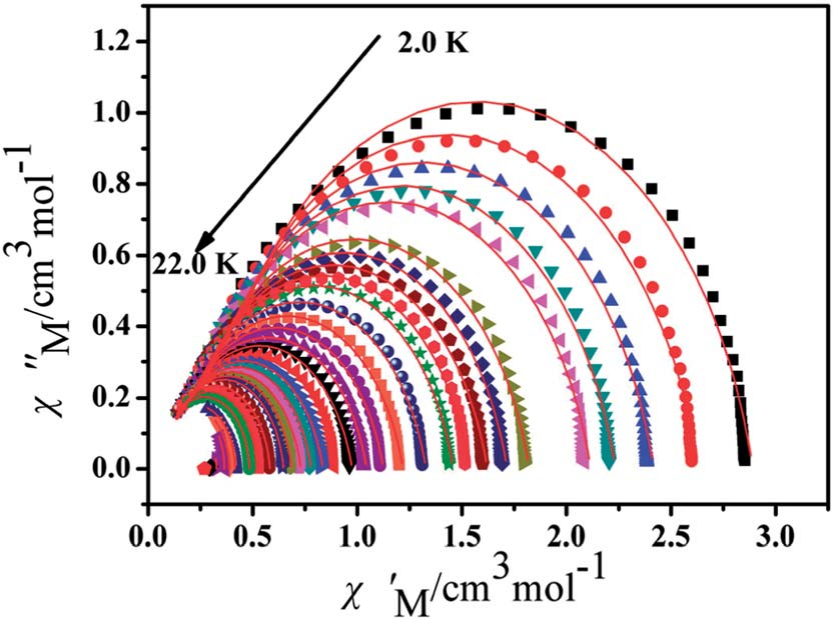
Cole–Cole plots for Cl-1 from 2.0 K to 22.0 K using the ac susceptibility data. The solid lines are the best fits for the generalized Debye model between 2.0 K and 22.0 K.

**Fig. 11:**
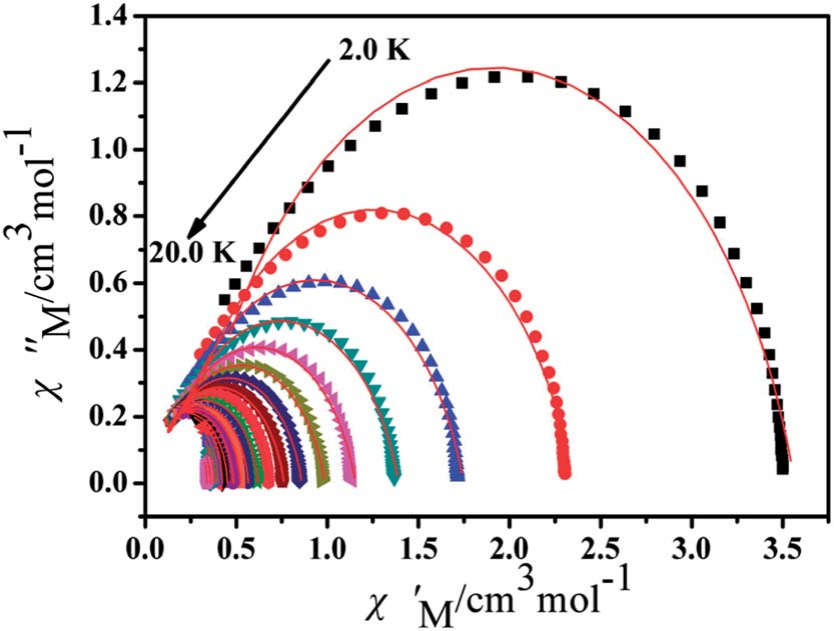
Cole–Cole plots for CH_3_O-2 from 2.0 K to 20.0 K using the ac susceptibility data. The solid lines are the best fits for the generalized Debye model between 2.0 K and 20.0 K.

**Fig. 12:**
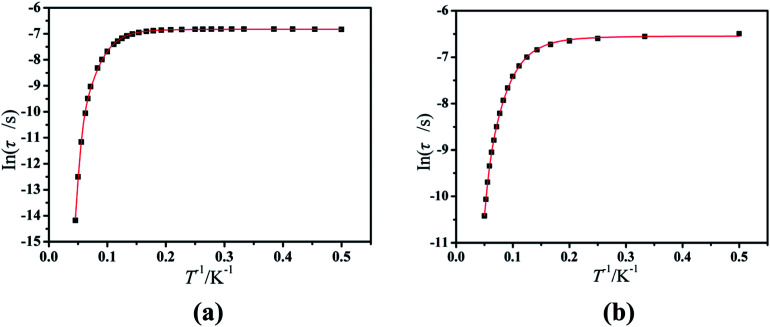
Magnetization relaxation time (ln(*τ*)) *versus T*^−1^ plots under a zero dc field for Cl-1 (a) and CH_3_O-2 (b). The solid red line represents the best fitting to the multiple relaxation processes (eqn (1), see text for parameters).

Furthermore, the frequency-dependent ac data for compounds Cl-1 and CH_3_O-2 were characterized in the absence of a dc field at various temperatures; the peaks of the *χ*′′ plots gradually shifted with the frequency sequence from middle to high, indicating that the *χ*′′ of compounds Cl-1 and CH_3_O-2 manifested frequency dependence in the selected temperature range (Fig. 8 and 9). The fitting of the Cole–Cole plots (*χ*′ M *vs. χ*′′) for Cl-1 and CH_3_O-2 (Fig. 10 and 11) with the Debye model^13^ presented a non-symmetric semicircle, which indicates the presence of a relatively moderate distribution of relaxation time (0.011 < *α* < 0.181 for Cl-1 and 0.007 < *α* < 0.161 for CH_3_O-2) (Table 3 and 4). For the relaxation time products under 0 Oe, the direct process can be neglected. The ln(*τ*) *versus* 1/*T* plots for compound Cl-1 and CH_3_O-2 presented some curvature (Fig. 12), indicating that the dynamics cannot be properly modelled by assuming a simple Orbach mechanism. Therefore, the total relaxation rates mainly reflect the Orbach process, Raman process and QTM process, using the following equation (eqn (1)):

1 *τ*^−1^ = *τ*_QTM_^−1^ + *CT*^*n*^ + *τ*_0_^−1^ exp(−*U*_eff_/*kT*)

where *τ* is the inverse of the ac frequency, *T* is the temperature of the maximum in the ac signal, *U*_eff_ is the effective energy barrier, *k* is Boltzmann's constant. *τ*_QTM_, *C*, and *τ*_0_ are the fitting parameters of the different relaxation mechanisms. In the absence of a static field, the independence of the relaxation time at low temperatures for compounds Cl-1 and CH_3_O-2 are indicative of QTM relaxation processes. The fit in the temperature range *T* = 2.0–22.0 K for compound Cl-1 by eqn (1) resulted in *τ*_QTM_ = 0.001 s, *n* = 4.97, *C* = 1.33 × 10^−3^ s^−1^ K^−4.97^, *τ*_0_ = 1.44 × 10^−8^ s, and an effective energy barrier of *U*_eff_ = 105.431 cm^−1^ (151.1 K). The fit in the temperature range *T* = 2.0–20.0 K for compound CH_3_O-2 by eqn (1) resulted in *τ*_QTM_ = 0.001 s, *n* = 4.06, *C* = 8.58 × 10^−2^ s^−1^ K^−4.06^, *τ*_0_ = 5.08 × 10^−9^ s, and an effective energy barrier of *U*_eff_ = 132.5 cm^−1^ (190.7 K).

**Table 2:** *Ab initio* computed relative energies (in cm^−1^), principal values of the g-tensors and averaged transition magnetic moments *μ*_QTM_ (in *β*) of the four lowest KD_s_ of the compounds studied in this work

		Cl-1	CH_3_O-2
KD_0_	*E*	0.000	0.000
	*g*_*Z*_	19.5623	19.5118
	*g*_*X*_	0.8671 × 10^−2^	0.1739 × 10^−2^
	*g*_*Y*_	0.1788 × 10^−1^	0.5135 × 10^−2^
	*g*_*XY*_	0.1987 × 10^−1^	0.5421 × 10^−2^
	*g*_*XY*_/*g*_*Z*_	0.1016 × 10^−2^	0.2779 × 10^−3^
	*μ*_QTM_	0.4425 × 10^−2^	0.1146 × 10^−2^
KD_1_	*E*	126.712 (105.431)^a^	152.849 (132.536)
	*g*_*Z*_	15.4850	15.1789
	*g*_*X*_	0.5304 × 10^+00^	0.8762 × 10^+00^
	*g*_*Y*_	0.1117 × 10^+01^	0.1462 × 10^+01^
	*g*_*XY*_	0.1237 × 10^+01^	0.1705 × 10^+01^
	*g*_*XY*_/*g*_*Z*_	0.7988 × 10^−1^	0.1123 × 10^+00^
	*μ*_QTM_	0.2746 × 10^+00^	0.3898 × 10^+00^
KD_2_	*E*	151.045	185.945
	*g*_*Z*_	17.6519	16.4613
	*g*_*X*_	0.4178 × 10^+00^	0.4877 × 10^+00^
	*g*_*Y*_	0.1481 × 10^+01^	0.2830 × 10^+01^
	*g*_*XY*_	0.1538 × 10^+01^	0.2872 × 10^+01^
	*g*_*XY*_/*g*_*Z*_	0.8716 × 10^−1^	0.1744 × 10^+01^
	*μ*_QTM_	0.3164 × 10^+00^	0.5530 × 10^+00^
KD_3_	*E*	177.793	251.832
	*g*_*Z*_	13.8539	2.5445
	*g*_*X*_	0.3264 × 10^+01^	0.8816 × 10^+01^
	*g*_*Y*_	0.3822 × 10^+01^	0.7151 × 10^+01^
	*g*_*XY*_	0.5026 × 10^+01^	0.1135 × 10^+02^
	*g*_*XY*_/*g*_*Z*_	0.3628 × 10^+00^	0.4461 × 10^+01^
	*μ*_QTM_	0.1181 × 10^+01^	0.2661 × 10^+01^

a Experimental *U*_eff_ is shown in parentheses.

**Table 3:** The results of preliminary ESP (in a.u.) analysis

	Cl-1	CH_3_O-2
ESP_(equ)_/ESP_(ax)_	0.723	0.701
ESP_(ax)_	1.082	1.162
ESP_(equ)_	0.783	0.814
ESP_(equ_N)_^a^	0.225	0.220
ESP_(equ_O)_^b^	0.557	0.594

a Contributions from the equatorial N atoms.

b Contributions from the equatorial O atoms.

The negative charges (in |*e*|) from *ab initio* calculations and the related Dy–O/N bond lengths (in Å) of the atoms in the first sphere^a^

**Table:** 

Cl-1	O_3_-ax	O_5_-ax	O_2_-ax	O_4_-ax	O_6_-equ	O_7_-equ	N_8_-equ	N_9_-equ
Charge	0.696	0.679	0.690	0.687	0.698	0.699	0.339	0.326
Dy–O/N	2.319 (7)	2.352 (6)	2.369 (6)	2.339 (6)	2.330 (6)	2.321 (6)	2.544 (8)	2.586 (8)

a “-ax” indicates the atoms at the axial positions and “-equ” means the atoms at equatorial positions.

**Table:** 

CH_3_O-2	O_2_-ax	O_7_-ax	O_3_-ax	O_5_-ax	O_4_-equ	O_6_-equ	N_8_-equ	N_9_-equ
Charge	0.722	0.764	0.704	0.710	0.764	0.697	0.332	0.304
Dy–O/N	2.350 (5)	2.307 (5)	2.318 (5)	2.305 (7)	2.337 (6)	2.312 (6)	2.554 (6)	2.571 (6)

The different local symmetries and the bond distances contribute to differing ligand fields, further resulting in diverse dynamic magnetic behaviors.^13*b*,35^ The CH_3_-4 (solvent) with an approximately square antiprismatic (SAP, *D*_4d_) N_2_O_6_ coordination environment exhibited more excellent properties than CH_3_-3 with a trigonal dodecahedron (*D*_2d_) configuration.^35^ However, the results in the present cases are in contrast to the explanation above, which verifies that the relaxation magnetism incompletely depends on the coordination symmetry of the Dy(iii) centers. Accordingly, the magnetism of dysprosium-based SMMs might be simultaneously dominated by complicated factors including local symmetry, electrostatics, *etc.*^10*b*,36^

In our recent work, a similar phenomenon was observed in the two β-diketone mononuclear Dy(iii) compounds, formulated as Dy(BTFA)_3_(H_2_O)_2_ (*D*_2d_) and Dy(BTFA)_3_(bpy) (*D*_4d_) (BTFA = 3-benzoyl-1,1,1-trifluoroacetone, bpy = 2,2′-bipyridine).^10*b*^ As noticed, compound Dy(BTFA)_3_(H_2_O)_2_ possesses a higher effective barrier than Dy(BTFA)_3_(bpy), despite Dy(BTFA)_3_(H_2_O)_2_ exhibiting a lower geometrical symmetry of the Dy(iii) ion. This is likely attributable to different charge distributions around the Dy(iii) ions in both compounds, which compensate for the discrepancy of the geometrical symmetries and is responsible for the disparities in magnetic anisotropy, as well as energy barrier and slow relaxation behavior between the two compounds. However, the conjecture above urgently needs studies for an in-depth understanding of the significative magneto-structural correlation.

According to the Dy(iii) coordination spheres, the compounds are slightly distorted, with the following order: F-5 > Cl-1 > CH_3_-4 (solvent); CH_3_-3 > CH_3_O-2. The shortest intermolecular distance between Dy(iii) ions is 7.911 (6) Å in Cl-1. However, there are no obvious ð–ð stacking or hydrogen bonding interactions in Cl-1, probably resulting in the weakening of the QTM relaxation process from the intermolecular interactions. CH_3_O-2 shows shorter bond lengths for Dy–N and Dy–O than CH_3_-3 and a smaller degree of distortion, indicating the strong charge density around the metal ions and further generating enhanced uniaxial magnetic anisotropy. Finally, these different effective energy barriers (Δ*E*/*k*_B_) have the following order: CH_3_O-2 > Cl-1 > CH_3_-4 (solvent) > CH_3_-3 > F-5.

### 3.3 Theoretical analysis

The effective energy barrier for the reversal of magnetization, *U*_eff_, is a popular parameter that is used to characterize the SMM properties of the compounds. However, only within the Orbach process is *U*_eff_ clearly defined in principle. In the early stages of the SMM, the compounds were usually polynuclear transition metal structures where the Orbach process dominated the magnetic relaxation; since then, *U*_eff_ has become popular in the field of SMM. However, in the case of mononuclear SIMs, several relaxation processes, including both Orbach and others of QTM, direct as well as Raman, exist simultaneously.^37–39^ Thus, the magnetic relaxation in Ln-based SIMs is not naturally dominated by the Orbach process and the necessary condition for the observation of SMM behavior is the effective suppression of all the fast relaxation process.^38,39^ Among all the fast relaxation processes, the quantum tunnelling of magnetization (QTM) within the ground state is the most effective and thus its suppression is the first target. Irrespective of various sources, the rate of QTM scales as the square of the so-called tunnel splitting Δ_tun_.^37–40^ For Kramers ions, *e.g.*, Dy(iii), *D*_tun_ is forced to be zero under the strict absence of a magnetic field due to time-reversal symmetry; however, small internal magnetic fields actually exist with different sources.^38^ Therefore Δ_tun_ in Kramers systems is induced *via* the Zeeman interaction (eqn (1a)) between the transversal fields (*H*_*X*_ and *H*_*Y*_) and the corresponding components of the magnetic moments μ⃗ of the same directions (*μ*_*X*_ and *μ*_*Y*_).^37–41^1a
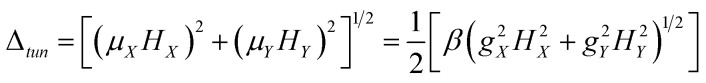
1b
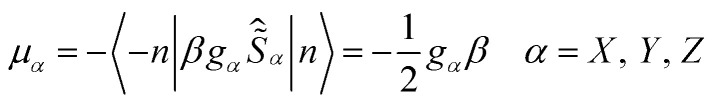
1c
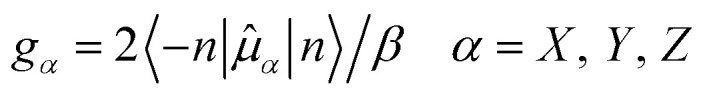


Theoretically, each Kramers doublet (KD) could be associated with an effective spin (pseudospin) *S* = 1/2.^28,37,38^ The magnetic moment of such pseudospin is determined by its principal values of the g-tensors as shown in eqn (1b).^37,38^ Clearly, small values of the transversal *g*_*X*_ and *g*_*Y*_ (eqn (1c)) of the ground KD, *i.e.*, KD_0_, will lead to a low magnitude of Δ_tun_ and thus zero-field SIM behavior could exist if the value of *g*_*XY*_ for KD_0_ is small enough.^42^ Besides the principal *g* values of each KD, *ab initio* calculations also provide the averaged absolute value of the transversal magnetic moments, *μ*_QTM_, which could also be used to measure the strength of QTM. As shown in Table 2, the *g*_*XY*_ values of KD_0_ are 0.1987 × 10^−1^ and 0.5421 × 10^−02^ for Cl-1 and CH_3_O-2, respectively. According to previous results from Ruiz *et al.*,^17^ zero-field SIM behavior could occur if the *g*_*XY*_ of KD_0_ is smaller than 0.15 × 10^−01^ for mononuclear Dy(iii) compounds. Clearly, this criterion is fulfilled in the case of CH_3_O-2 and, although a little bit larger, the *g*_*XY*_ of KD_0_ of Cl-1 is also quite close to this value. Therefore our *ab initio* results do suggest the existence of zero-field SIM properties in these two compounds, which is consistent with the experimental observation based on ac susceptibility measurements.

Due to the smaller value of *g*_*XY*_ of KD_0_, the SIM property of CH_3_O-2 is theoretically predicted to be superior to that of Cl-1. This theoretical prediction is also in line with the higher *U*_eff_ of CH_3_O-2 obtained from the fitting of the experimental data. In many cases of Ln-SIMs, the energy of the first excited KD, *i.e.*, KD_1_, is closely related to the *U*_eff_. In the two compounds here, the energies of KD_1_ are indeed close to the experimental *U*_eff_: 126.7 cm^−1^*vs.* 105.4 cm^−1^ for Cl-1 and 152.8 cm^−1^*vs.* 132.5 cm^−1^ for CH_3_O-2. Moreover, the energy of KD_1_ for CH_3_O-2 is also higher than that of Cl-1. Thus, the reliability of our *ab initio* results is verified again in terms of energies of KD_1_.

**Fig. 13:**
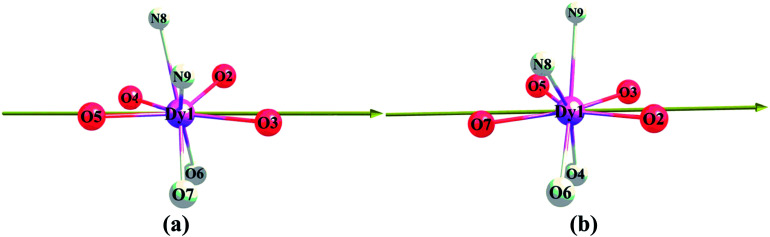
Direction of the *ab initio* magnetic easy axis of the ground KD_s_ of the compounds (only the central Dy(iii) and first sphere are shown for the sake of clarity, the equatorial atoms are shaded).

As shown in our previous results,^14,18,19^ the desired electronic structure, which is suitable for the ideal SIM properties of Dy(iii) systems, could be approached *via* an electrostatic route due to the oblate electron density of the Dy(iii) ion; *i.e.*, the axial electrostatic potential (ESP) should exceed the equatorial one as much as possible.^6*g*,31,40,43^ According to the orientation of the magnetic easy axis (Fig. 13), the eight atoms of the first sphere could be collected into two groups: (1) axial atoms consisting of the four oxygen atoms (O2, O3, O4 and O5 for Cl-1) that lie along the axial direction; (2) equatorial atoms consisting of the two nitrogen atoms and the residual two oxygen atoms (N8, N9, O6 and O7 for Cl-1). With *ab initio* atomic charge, we could approximate the axial ESP felt by the Dy(iii) ion, *i.e.*, ESP_(ax)_, with the sum of the contribution from the four axial atoms. Similarly, the equatorial ESP, *i.e.*, ESP_(equ)_, could be approximated as the collection of the contribution from the four equatorial atoms. As shown before,^31,40,43^ the lower value of the ratio ESP_(equ)_/ESP_(ax)_ indicates the higher degree of the excess of the axial ESP over the equatorial, and thus it should lead to the electronic structure, which is more suitable for the ideal SIM properties.

As shown in Table 3, the ESP_(equ)_/ESP_(ax)_ ratio of CH_3_O-2 is 0.701, clearly lower than that of Cl-1 (0.723). Thus, the superior SIM property of CH_3_O-2 should originate from the more suitable ESP felt by the central Dy(iii) ion when compared with 1. In detail, the difference in ESP_(equ)_ of these two compounds is 0.03 a.u., which is clearly smaller than the corresponding difference in ESP_(ax)_ (0.08 a.u.). In other words, the different amounts of ESP_(ax)_ of these two compounds should play the central role in their differences in terms of SIM properties. When making a further analysis of the charges and distances to the central ion (Table 4), the averaged charges and distances to the central Dy(iii) ion are 0.725 |*e*| and 2.320 Å, respectively, for CH_3_O-2. In the case of Cl-1, the averaged charges and distances are 0.688 |*e*| and 2.345 Å, respectively. Therefore the larger amount of ESP_(ax)_ for CH_3_O-2 arises from the synergic effect of the more negative charge and shorter Dy–O distances of the axial O atoms of the first sphere. Of course, these charges and distances could be influenced by functional groups outside the first sphere, *e.g.*, –Cl and –OCH_3_.

## 4. Conclusion

Two mononuclear compounds, [Dy(Phen)(Cl-tcpb)_3_] (Cl-1) and [Dy(Phen)(CH_3_O-tmpd)_3_] (CH_3_O-2), were synthesized based on β-diketonate ligands (Cl-tcpb = 1-(4-chlorophenyl)-4,4,4-trifluoro-1,3-butanedione and CH_3_O-tmpd = 4,4,4-trifluoro-1-(4-methoxyphenyl)-1,3-butanedione) with different substituent groups (–Cl/–OCH_3_) and auxiliary ligand 1,10-phenanthroline (Phen). The Dy(iii) ions in Cl-1 have approximately square-antiprismatic (SAP, *D*_4d_) N_2_O_6_ coordination environments. The coordination geometry of Dy(iii) ions in CH_3_O-2 can be best described as a trigonal dodecahedron (*D*_2d_). The dynamic magnetic investigations showed that both compounds exhibited SMM behavior in zero dc field, while the effective magnetization relaxation barriers increased progressively from 105.4 cm^−1^ (151.1 K) for Cl-1 to 132.5 cm^−1^ (190.7 K) for CH_3_O-2. CH_3_O-2 possessed a higher effective barrier than Cl-1, despite Cl-1 exhibiting a higher geometrical symmetry of the Dy(iii) ion. Moreover, the energy of KD_1_ of CH_3_O-2 was also higher than that of Cl-1. The zero-field SIM behaviors, as well as the difference in *U*_eff_, of these two compounds were reproduced by *ab initio* calculations. Further studies from the viewpoint of electrostatic potential demonstrated that the larger axial electrostatic potential (ESP) felt by the central Dy(iii) ion of CH_3_O-2 is responsible for its better SIM properties when compared with Cl-1. The larger amount of ESP_(ax)_ of CH_3_O-2 arises from the synergic effect of the more negative charge and shorter Dy–O distances of the axial O atoms of the first sphere. Beyond all doubt, these charges and distances could be affected by functional groups outside the first sphere, *e.g.*, –Cl and –OCH_3_.

## Conflicts of interest

There are no conflicts to declare.

## Supplementary Material

RA-008-C8RA06240D-s001

RA-008-C8RA06240D-s002
