# Supplementary material for: Experimental and theoretical interpretation of the magnetic behavior of two Dy(iii) single-ion magnets constructed through β-diketonate ligands with different substituent groups (–Cl/–OCH3)
Source: RSC Adv. 2018 Aug 20;8(52):29513–25. doi: 10.1039/c8ra06240d (PMC9092431; doi:10.1039/c8ra06240d)
Supplement: RA-008-C8RA06240D-s001 [file RA-008-C8RA06240D-s001.pdf]

Electronic Supplementary Material (ESI) for RSC Advances.
This journal is © The Royal Society of Chemistry 2018

Experimental and Theoretical Interpretation on the Magnetic Behavior for Two Dy (III) Single-Ion Magnets Constructed Through *β*-Diketonate Ligands with Different Substituent Groups (-Cl/-OCH_3)

Sheng Zhang,[a,c*] Wenjiao Mo,[a] Jiangwei Zhang,[d] Haipeng Wu,[b] Min Li,[b] Xingqiang Lü,[c] Bing Yin,[b*] Desuo Yang [a*]

[a.] *College of Chemistry and Chemical Engineering, Baoji University of Arts and Sciences, Baoji 721013, China.*

[b.] *Key Laboratory of Synthetic and Natural Functional Molecule Chemistry of Ministry of Education, College of Chemistry and Materials Science, Northwest University, Xi'an, Shaanxi 710069, China.*

[c.] *School of Chemical Engineering, Shaanxi Key Laboratory of Degradable Medical Material, Northwest University, Xi'an 710069, Shaanxi, China*

[d.] *State Key Laboratory of Catalysis & Gold Catalysis Research Center,Dalian Institute of Chemical Physics, Chinese Academy of Sciences (CAS)*

Table S1 Crystal data and structure refinement details for **Cl-1** and **CH_3O-2**.

Compound	**Cl-1**	**CH_3O-2**
Empirical formula	C42H23Cl3DyF9N2O6	C45H32DyF9N2O9
CCDC	1815606	1815605
T (K)	293 (2)	130 (2)
Formula weight	1091.47	1078.23
Crystal system	Triclinic	Triclinic
Space group	*p-1*	*p-1*
a/Å	11.5704(11)	10.7420(16)
b/Å	12.1367(11)	12.7566(17)
c/Å	15.4949(13)	16.960(2)
α/°	98.652(7)	85.703(11)
β/°	100.231(8)	80.218(12)
γ/°	102.305(8)	66.118(13)
V/Å^3	2051.6(3)	2094.1(5)
Z	2	2
$\rho_{calc.}$/g·cm^{-3}	1.767	1.710
μ/mm^{-1}	12.367	10.442
F(000)	1070	1070
θ min-max/°	4.335 to 59.999	3.922 to 68.524
Total reflections	11224	14387

Unique reflections	6059	7479
Goodness-of-fit on F^2	1.008	1.027
R_1/wR_2 [$I>2\sigma(I)$]	0.0639 / 0.0855	0.0635/ 0.1479
R_1/wR_2 [all data]	0.1255 / 0.1123	0.0961 / 0.1863
Largest peak and hole/ e Å$^-$	0.970 / -0.765	0.564 / -0.727

* $R_1 = \Sigma\|F_0|-|F_c\|/\Sigma|F_0|$, $wR_2 = [\Sigma(F_0^2 - F_c^2)/\Sigma w(F_0)^2]^{1/2}$, $w = [\sigma_c^2(F_0^2) + (xP)^2 + yP]^{-1}$, $P = (F_0^2 + 2F_c^2)/3$

Table S2 Selected bond lengths (Å) and angles (°) for **Cl-1** and **CH_3O-2**.

**Cl-1**			
Dy(1)-O(2)	2.319(7)	O(2)-Dy(1)-O(1)	72.6(2)
Dy(1)-O(6)	2.321(6)	O(6)-Dy(1)-O(1)	138.2(2)
Dy(1)-O(5)	2.330(6)	O(5)-Dy(1)-O(1)	77.2(2)
Dy(1)-O(3)	2.339(6)	O(3)-Dy(1)-O(1)	72.9(2)
Dy(1)-O(4)	2.352(6)	O(4)-Dy(1)-O(1)	139.1(2)
Dy(1)-O(1)	2.369(6)	O(2)-Dy(1)-N(1)	105.5(2)
Dy(1)-N(1)	2.544(8)	O(6)-Dy(1)-N(1)	138.8(3)
Dy(1)-N(2)	2.586(8)	O(5)-Dy(1)-N(1)	147.3(2)
O(2)-Dy(1)-O(6)	75.8(2)	O(3)-Dy(1)-N(1)	77.4(3)
O(2)-Dy(1)-O(5)	85.1(2)	O(4)-Dy(1)-N(1)	77.3(3)
O(6)-Dy(1)-O(5)	73.4(2)	O(1)-Dy(1)-N(1)	76.8(2)
O(2)-Dy(1)-O(3)	143.6(2)	O(2)-Dy(1)-N(2)	77.3(2)
O(6)-Dy(1)-O(3)	126.1(2)	O(6)-Dy(1)-N(2)	76.8(2)
O(5)-Dy(1)-O(3)	76.3(2)	O(5)-Dy(1)-N(2)	148.3(2)
O(2)-Dy(1)-O(4)	145.5(2)	O(3)-Dy(1)-N(2)	131.8(3)
O(6)-Dy(1)-O(4)	80.2(2)	O(4)-Dy(1)-N(2)	73.4(2)
O(5)-Dy(1)-O(4)	111.4(2)	O(1)-Dy(1)-N(2)	120.8(2)
O(3)-Dy(1)-O(4)	70.9(2)	N(1)-Dy(1)-N(2)	63.9(3)

**CH_3O-2**			
Dy(1)-O(1)	2.305(7)	O(1)-Dy(1)-N(1)	78.9(2)
Dy(1)-O(5)	2.307(5)	O(5)-Dy(1)-N(1)	150.0(2)
Dy(1)-O(8)	2.312(6)	O(8)-Dy(1)-N(1)	130.0(2)
Dy(1)-O(2)	2.318(5)	O(2)-Dy(1)-N(1)	121.0(2)
Dy(1)-O(7)	2.337(6)	O(7)-Dy(1)-N(1)	80.8(2)
Dy(1)-O(4)	2.350(5)	O(4)-Dy(1)-N(1)	78.28(19)
Dy(1)-N(1)	2.554(6)	O(1)-Dy(1)-N(2)	103.3(2)
Dy(1)-N(2)	2.571(6)	O(5)-Dy(1)-N(2)	146.0(2)
O(1)-Dy(1)-O(5)	88.0(2)	O(8)-Dy(1)-N(2)	75.4(2)
O(1)-Dy(1)-O(8)	140.71(19)	O(2)-Dy(1)-N(2)	75.06(19)
O(5)-Dy(1)-O(8)	75.76(19)	O(7)-Dy(1)-N(2)	90.3(2)
O(1)-Dy(1)-O(2)	71.2(2)	O(4)-Dy(1)-N(2)	141.19(18)

O(5)-Dy(1)-O(2)	78.70(19)	N(1)-Dy(1)-N(2)	63.9(2)
O(8)-Dy(1)-O(2)	70.6(2)	O(1)-Dy(1)-N(1)	78.9(2)
O(1)-Dy(1)-O(7)	147.2(2)	O(5)-Dy(1)-N(1)	150.0(2)
O(5)-Dy(1)-O(7)	97.1(2)	O(8)-Dy(1)-N(1)	130.0(2)
O(8)-Dy(1)-O(7)	71.31(19)	O(2)-Dy(1)-N(1)	121.0(2)
O(2)-Dy(1)-O(7)	141.6(2)	O(7)-Dy(1)-N(1)	80.8(2)
O(1)-Dy(1)-O(4)	76.2(2)	O(4)-Dy(1)-N(1)	78.28(19)
O(5)-Dy(1)-O(4)	72.39(18)	O(1)-Dy(1)-N(2)	103.3(2)
O(8)-Dy(1)-O(4)	129.3(2)	O(5)-Dy(1)-N(2)	146.0(2)
O(2)-Dy(1)-O(4)	136.71(19)	O(8)-Dy(1)-N(2)	75.4(2)
O(7)-Dy(1)-O(4)	74.7(2)	O(2)-Dy(1)-N(2)	75.06(19)
O(8)-Dy(1)-N(1)	130.0(2)	O(7)-Dy(1)-N(2)	90.3(2)
O(2)-Dy(1)-N(1)	121.0(2)	O(4)-Dy(1)-N(2)	141.19(18)
O(7)-Dy(1)-N(1)	80.8(2)	N(1)-Dy(1)-N(2)	63.9(2)
O(4)-Dy(1)-N(1)	78.28(19)	O(1)-Dy(1)-N(1)	78.9(2)
O(1)-Dy(1)-N(2)	103.3(2)	O(5)-Dy(1)-N(1)	150.0(2)

Table S3 Dy^{III} ion geometry analysis by SHAPE 2.1 software.

Configuration	ABOXIY, **Cl-1**	ABOXIY, **CH_3O-2**	ABOXIY, **CH_3-3**	ABOXIY, **CH_3-4 (solvent)**	ABOXIY, **F-5**
Hexagonal bipyramid (D_{6h})	15.904	15.904	15.235	16.278	15.550
Cube (O_h)	10.507	9.035	8.971	9.645	10.541
Square antiprism (D_{4d})	0.791	1.888	1.520	0.484	1.344
Triangular dodecahedron (D_{2d})	1.341	0.621	0.792	2.106	1.509
Johnson gyrobifastigium J26 (D_{2d})	14.375	14.958	14.859	15.939	14.497
Johnson elongated triangular bipyramid J14 (D_{3h})	27.695	29.352	29.178	27.903	28.104
Biaugmented trigonal prism J50 (C_{2v})	2.296	2.743	2.404	2.376	1.785
Biaugmented trigonal prism (C_{2v})	1.935	1.957	1.792	1.737	1.314
Snub siphenoid J84 (D_{2d})	3.732	3.565	3.429	4.992	4.070

Cl-1

HBPY-8 3 D6h Hexagonal bipyramid
CU-8 4 Oh Cube
SAPR-8 5 D4d Square antiprism
TDD-8 6 D2d Triangular dodecahedron
JGBF-8 7 D2d Johnson gyrobifastigium J26
JETBPY-8 8 D3h Johnson elongated triangular bipyramid J14
JBTPR-8 9 C2v Biaugmented trigonal prism J50
BTPR-8 10 C2v Biaugmented trigonal prism
JSD-8 11 D2d Snub diphenoid J84

Structure [ML8] HBPY-8 CU-8 SAPR-8 TDD-8 JGBF-8 JETBPY-8 JBTPR-8 BTPR-8 JSD-8
ABOXIY , 16.745, 10.507, 0.791, 1.341, 14.375, 27.695, 2.296, 1.935, 3.732

CH_3O-2

HBPY-8	3 D6h	Hexagonal bipyramid
CU-8	4 Oh	Cube
SAPR-8	5 D4d	Square antiprism
TDD-8	6 D2d	Triangular dodecahedron
JGBF-8	7 D2d	Johnson gyrobifastigium J26
JETBPY-8	8 D3h	Johnson elongated triangular bipyramid J14
JBTPR-8	9 C2v	Biaugmented trigonal prism J50
BTPR-8	10 C2v	Biaugmented trigonal prism
JSD-8	11 D2d	Snub diphenoid J84

Structure [ML8]		HBPY-8	CU-8	SAPR-8	TDD-8	JGBF-8	JETBPY-8	JBTPR-8	BTPR-8	JSD-8
ABOXIY	,	15.904,	9.035,	1.888,	0.621,	14.958,	29.352,	2.743,	1.957,	3.565

CH_3-3

HBPY-8	3 D6h	Hexagonal bipyramid
CU-8	4 Oh	Cube
SAPR-8	5 D4d	Square antiprism
TDD-8	6 D2d	Triangular dodecahedron
JGBF-8	7 D2d	Johnson gyrobifastigium J26
JETBPY-8	8 D3h	Johnson elongated triangular bipyramid J14
JBTPR-8	9 C2v	Biaugmented trigonal prism J50
BTPR-8	10 C2v	Biaugmented trigonal prism
JSD-8	11 D2d	Snub diphenoid J84

Structure [ML8]		HBPY-8	CU-8	SAPR-8	TDD-8	JGBF-8	JETBPY-8	JBTPR-8	BTPR-8	JSD-8
ABOXIY	,	15.235,	8.971,	1.520,	0.792,	14.859,	29.178,	2.404,	1.792,	3.429

CH_3-4 (solvent)

HBPY-8	3 D6h	Hexagonal bipyramid
CU-8	4 Oh	Cube
SAPR-8	5 D4d	Square antiprism
TDD-8	6 D2d	Triangular dodecahedron
JGBF-8	7 D2d	Johnson gyrobifastigium J26
JETBPY-8	8 D3h	Johnson elongated triangular bipyramid J14
JBTPR-8	9 C2v	Biaugmented trigonal prism J50
BTPR-8	10 C2v	Biaugmented trigonal prism
JSD-8	11 D2d	Snub diphenoid J84

Structure [ML8]		HBPY-8	CU-8	SAPR-8	TDD-8	JGBF-8	JETBPY-8	JBTPR-8	BTPR-8	JSD-8
ABOXIY	,	16.278,	9.645,	0.484,	2.106,	15.939,	27.903,	2.376,	1.737,	4.992

F-5

HBPY-8	3 D6h	Hexagonal bipyramid
CU-8	4 Oh	Cube
SAPR-8	5 D4d	Square antiprism
TDD-8	6 D2d	Triangular dodecahedron
JGBF-8	7 D2d	Johnson gyrobifastigium J26
JETBPY-8	8 D3h	Johnson elongated triangular bipyramid J14
JBTPR-8	9 C2v	Biaugmented trigonal prism J50
BTPR-8	10 C2v	Biaugmented trigonal prism
JSD-8	11 D2d	Snub diphenoid J84

Structure [ML8]		HBPY-8	CU-8	SAPR-8	TDD-8	JGBF-8	JETBPY-8	JBTPR-8	BTPR-8	JSD-8
ABOXIY	,	15.550,	10.541,	1.344,	1.509,	14.497,	28.104,	1.785,	1.314,	4.070

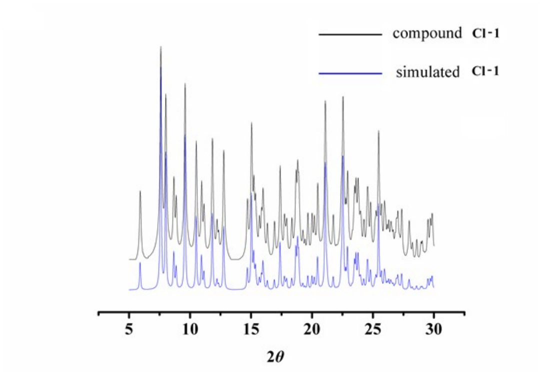

(a)

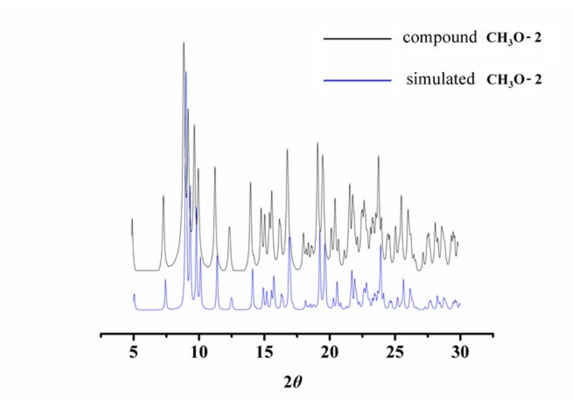

(b)

Fig. S1 XRPD curves of **Cl-1** (a) and **CH_3O-2** (b).

Table S4 Relaxation fitting parameters from Least-Squares Fitting of $\chi(\omega)$ data of **Cl-1**.

$\Delta\chi_1$ (cm^3mol^{-1})	$\Delta\chi_2$ (cm^3mol^{-1})	τ_0(s)	α_1	Residual
0.260267	2.88972	0.00108023	0.152772	0.0544879
0.235467	2.63226	0.00108632	0.154364	0.0448944
0.215929	2.41606	0.00109073	0.154924	0.0387642
0.198845	2.23348	0.00109272	0.155788	0.0334581
0.185081	2.11559	0.00109064	0.159417	0.0315545
0.161256	1.82153	0.00109452	0.158183	0.0230387
0.151712	1.71551	0.00109334	0.158845	0.0203543
0.143558	1.62042	0.00109116	0.159116	0.018353
0.135971	1.53591	0.00108856	0.159742	0.016524
0.129317	1.45934	0.00108448	0.159882	0.0150249
0.117977	1.32793	0.00107534	0.160704	0.0121908
0.109049	1.2178	0.00106267	0.160384	0.0103158
0.101474	1.12452	0.00104546	0.159654	0.0087332
0.0952804	1.04441	0.00102376	0.157997	0.00738076
0.0904192	0.974851	0.000996952	0.154781	0.0063388
0.0855923	0.899736	0.000954786	0.148749	0.00537565
0.0816003	0.83514	0.000901332	0.140304	0.00446558
0.0781279	0.779001	0.000837319	0.129569	0.00360929
0.0749899	0.729782	0.000764925	0.117124	0.00286337
0.0722477	0.686123	0.000687403	0.103531	0.00223526
0.0693756	0.647638	0.000608463	0.0905437	0.00161829
0.0642372	0.58199	0.000460389	0.0662283	0.000804666
0.0600902	0.528562	0.000338316	0.0462514	0.000370617
0.0568107	0.484583	0.000244763	0.03203	0.000155266
0.0502608	0.415816	0.000119337	0.0144383	3.37361E-05
0.0347966	0.388734	7.53425E-05	0.0125559	1.89366E-05
2.23268E-09	0.365537	4.27867E-05	0.0111428	1.64302E-05

Table S5 Relaxation fitting parameters from Least-Squares Fitting of $\chi(\omega)$ data of **CH_3O-2**.

$\Delta\chi_1$ (cm^3mol^{-1})	$\Delta\chi_2$ (cm^3mol^{-1})	τ_0(s)	α_1	Residual
0.266507	3.56355	0.001515	0.176318	0.110833
0.168186	2.34622	0.001424	0.17889	0.052804
0.119823	1.74644	0.001363	0.1812	0.031467
0.091869	1.3902	0.001296	0.180892	0.020606
0.075094	1.1541	0.0012	0.175946	0.014766
0.064386	0.985479	0.001067	0.166665	0.011127
0.055593	0.859953	0.000912	0.154186	0.008216
0.048402	0.762001	0.000753	0.137986	0.006132
0.042738	0.683631	0.000603	0.120175	0.004326
0.037046	0.61968	0.00047	0.101782	0.002898
0.031611	0.56649	0.00036	0.083411	0.001791
0.022852	0.522052	0.000272	0.068876	0.000984
0.012178	0.484203	0.000204	0.054569	0.000493
4.46E-14	0.451358	0.000152	0.039521	0.000264
1.22E-13	0.423316	0.000118	0.013788	0.000167
